# Deleterious Alleles in the Human Genome Are on Average Younger Than Neutral Alleles of the Same Frequency

**DOI:** 10.1371/journal.pgen.1003301

**Published:** 2013-02-28

**Authors:** Adam Kiezun, Sara L. Pulit, Laurent C. Francioli, Freerk van Dijk, Morris Swertz, Dorret I. Boomsma, Cornelia M. van Duijn, P. Eline Slagboom, G. J. B. van Ommen, Cisca Wijmenga, Genome of the Netherlands Consortium, Paul I. W. de Bakker, Shamil R. Sunyaev

**Affiliations:** 1 Division of Genetics, Department of Medicine, Brigham and Women's Hospital, Harvard Medical School, Boston, Massachusetts, United States of America; 2 Program in Medical and Population Genetics, Broad Institute of MIT and Harvard, Cambridge, Massachusetts, United States of America; 3 Cancer Program, Broad Institute of MIT and Harvard, Cambridge, Massachusetts, United States of America; 4 Departments of Medical Genetics and of Epidemiology, University Medical Center Utrecht, Utrecht, The Netherlands; 5 Department of Genetics, University of Groningen, University Medical Center Groningen, Groningen, The Netherlands; 6 Department of Biological Psychology, VU University, Amsterdam, The Netherlands; 7 Department of Epidemiology, Erasmus Medical Center, Rotterdam, The Netherlands; 8 Department of Medical Statistics and Bioinformatics, Leiden University Medical Center, Leiden, The Netherlands; 9 Department of Human Genetics, Leiden University Medical Center, Leiden, The Netherlands; University of Wisconsin–Madison, United States of America

## Abstract

Large-scale population sequencing studies provide a complete picture of human genetic variation within the studied populations. A key challenge is to identify, among the myriad alleles, those variants that have an effect on molecular function, phenotypes, and reproductive fitness. Most non-neutral variation consists of deleterious alleles segregating at low population frequency due to incessant mutation. To date, studies characterizing selection against deleterious alleles have been based on allele frequency (testing for a relative excess of rare alleles) or ratio of polymorphism to divergence (testing for a relative increase in the number of polymorphic alleles). Here, starting from Maruyama's theoretical prediction (Maruyama T (1974), Am J Hum Genet USA 6:669–673) that a (slightly) deleterious allele is, on average, younger than a neutral allele segregating at the same frequency, we devised an approach to characterize selection based on allelic age. Unlike existing methods, it compares sets of neutral and deleterious sequence variants at the same allele frequency. When applied to human sequence data from the Genome of the Netherlands Project, our approach distinguishes low-frequency coding non-synonymous variants from synonymous and non-coding variants at the same allele frequency and discriminates between sets of variants independently predicted to be benign or damaging for protein structure and function. The results confirm the abundance of slightly deleterious coding variation in humans.

## Author Summary

A key challenge in human genetics is to identify, among the multitude of genetic differences between individuals, those that have an effect on traits. Even though new genetic variants arise through mutation in each generation, most are present only in a small proportion of individuals because they have slightly negative effects on fitness. Detecting such slightly deleterious variants is a key challenge in analyzing how genetics influence human characteristics. In this paper, we test a theoretical prediction by Takeo Maruyama from 1974 that a slightly deleterious variant is, on average, younger than a neutral (non affecting fitness) variant present at the same population frequency. Thus our method detects selection by using estimated age of variants. We applied our method to human data from the Genome of the Netherlands Project, and we show that it distinguishes low-frequency protein-modifying variants from silent variants at the same population frequency and discriminates between sets of variants predicted to be benign or damaging for protein structure and function. Our results confirm the abundance of slightly deleterious protein-coding variation in humans.

## Introduction

Most studies of deleterious genetic variation in humans have focused on the allele frequency spectrum and on the excess of rare alleles at functionally significant sites [1]–[7]. However, information about a deleterious effect of an allele is not limited to its population frequency. A classic result by Takeo Maruyama [8] predicts that both deleterious and advantageous alleles are younger (arose more recently by mutation events) than neutral alleles at the same population frequency. The predicted difference in age is greater for more strongly selected alleles. Intuitively, a deleterious allele is less likely to reach a given population frequency than a neutral allele. However, if it does reach this frequency, it likely did so in a short sequence of steps.

Under the assumption of constant population size and no dominance, mean allelic age conditional on population frequency is exactly symmetric with respect to direction of selection — beneficial and deleterious alleles with the same absolute value of the selection coefficient at the same frequency have identical mean ages.

Thus, a profound consequence of Maruyama's theoretical prediction is that it enables statistical discrimination between classes of neutral and deleterious alleles even if the alleles are at the same population frequency. Approximating allelic age conditional on present allele frequency may provide a new way to quantify deleterious genetic variation, independent from analyses based on allele frequency distribution or polymorphism-to-divergence ratio. Conditional on current allele frequency, both allelic age and, in particular, time spent in the past at higher frequencies can be estimated by enumerating either mutation or recombination events after the first appearance of the allele in the population. Approaches based on comparison of allelic ages have been previously used to detect alleles under positive selection [9]–[11]. The same basic principle can be extended to the analysis of deleterious variation. We have taken this idea to characterize deleterious variation in sequencing data.

Some existing methods for estimating age use intra-allelic variability [12], patterns of linkage disequilibrium [13], or shared haplotypes [14]. These approaches were designed for fine-mapping of mutations or for estimating the absolute age of very rare mutations and may therefore be unsuitable for genome-wide analyses. Importantly, as we show below, difference in sojourn times at higher frequencies is more informative than the allelic age. Therefore, a statistical approach based on comparison of sojourn times at higher frequencies is potentially more powerful than an approach based on the estimation of the allelic age.

Here, using a new dataset of completely sequenced parent-child trios, we provide evidence that the “Maruyama effect” (i.e., at a given allele frequency, deleterious alleles are on average younger than neutral ones) can be observed in human genetic data. We introduce a statistic that is based on proximity of completely linked mutations at a lower frequency and recombination events. We demonstrate that this statistic can successfully discriminate between functional classes of human low-frequency derived allelic variants even if they are at the same frequency. This confirms the abundant selection against deleterious alleles in the human population.

## Results/Discussion

**Figure 1:**
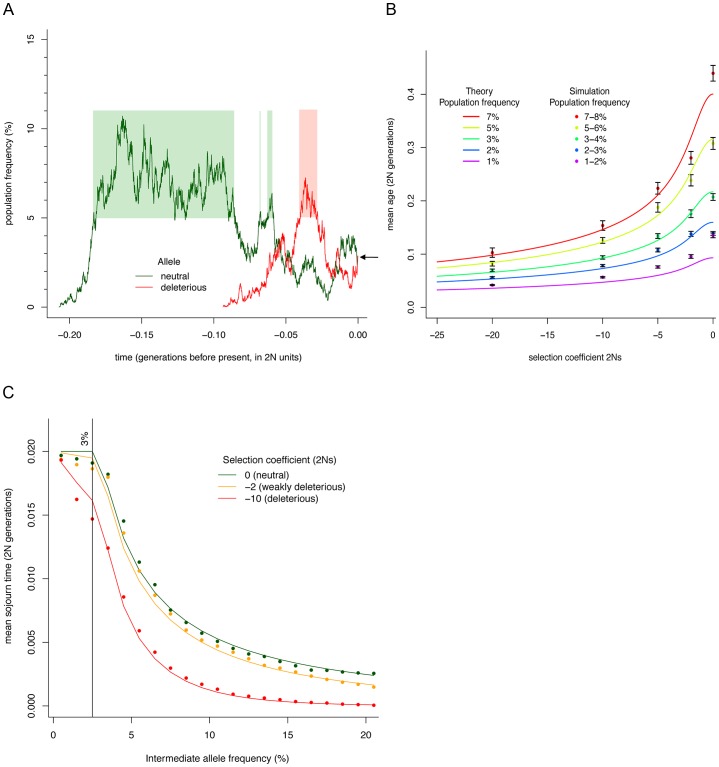
Simulation and theoretical results for allelic age and sojourn times. a. Example trajectories for a neutral and deleterious allele with current population frequencies 3% (indicated by the arrow). The shaded areas indicate sojourn times at frequencies above 5%. b. Mean ages for neutral and deleterious alleles at a given population frequency (lines show theoretical predictions, dots show simulation results with standard error bars). Simulation results are averages of alleles in a frequency range, while theoretical prediction are for alleles at a fixed frequency. The graph shows that deleterious alleles at a given frequency are younger than neutral alleles, and that the effect is greater for more strongly selected alleles. c. Mean sojourn times for neutral and deleterious alleles. Vertical line denotes the current population frequency of the variant (3%). Mean sojourn times have been computed in bins of 1%. Line connects theoretical predictions for each frequency bin. Dots show simulation results. The graph illustrates that deleterious alleles spend much less time than neutral alleles at higher population frequencies in the past even if they have the same current frequency.

First, we recapitulated Maruyama's theory with diffusion approximation and simulations (see Methods) and confirm that neutral alleles at a given frequency are older than selected ones (Figure 1a, 1b). A neutral allele observed at frequency 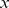 spent, on average, an equal amount of time at each frequency below 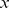, whereas a deleterious allele spent progressively shorter time at higher frequencies (Figure 1c). The difference in the average age of neutral and selected alleles is primarily due to shorter sojourn times at higher frequencies for selected alleles. This suggests that a statistic capturing sojourn times at higher frequencies would better discriminate between neutral and selected alleles than a statistic based on an accurate estimation of the allelic age.

**Figure 2:**
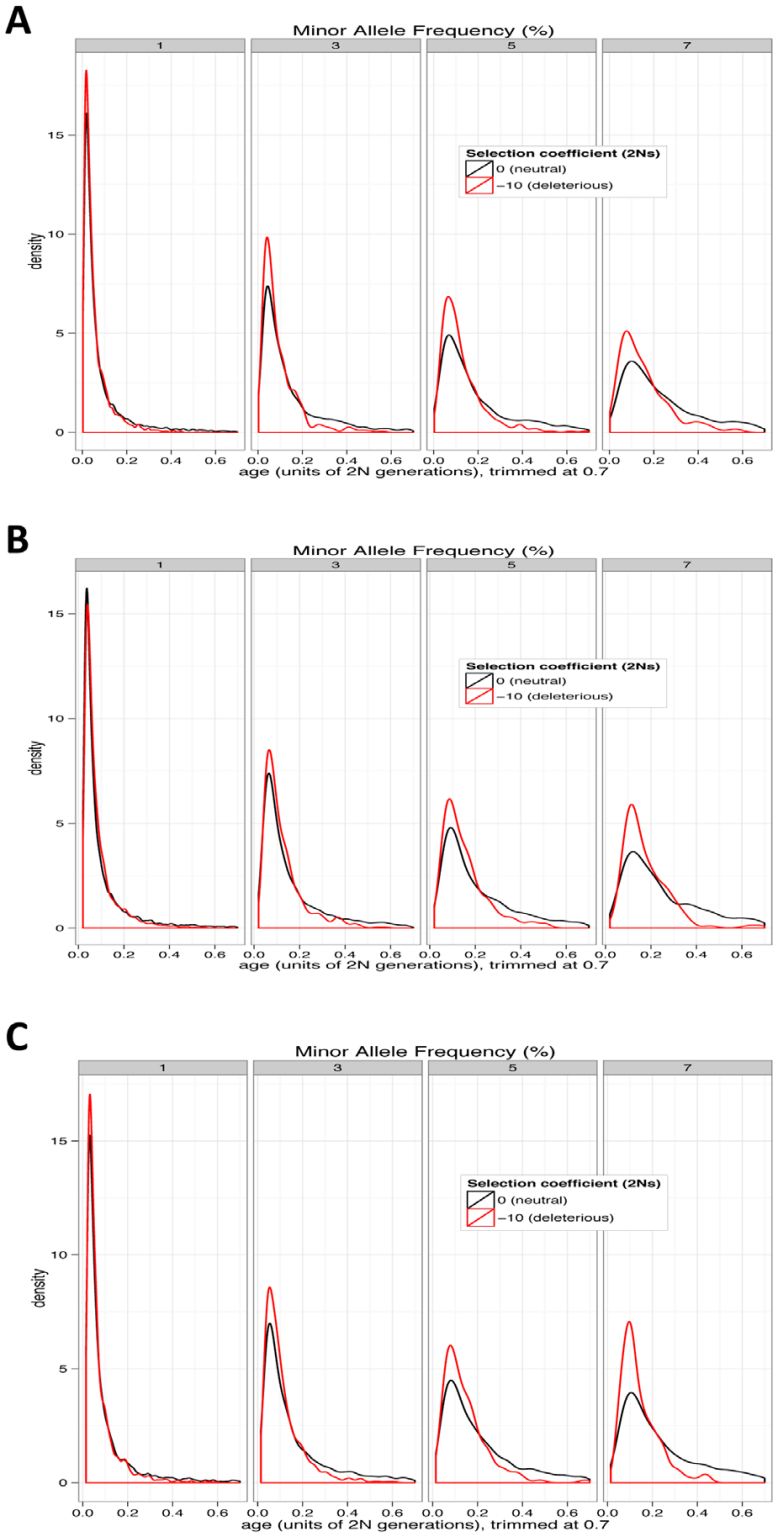
Age distributions for neutral and deleterious alleles from simulations. (A) Constant-size, (B) recently rapidly expanding population, and (C) bottleneck followed by rapid expansion. For presentation, distributions are trimmed. Deleterious alleles in all cases are younger than neutral alleles at the same frequency, though the effect is weaker in rapidly expanding populations.

Both mean allelic age and mean sojourn times at each frequency are exactly symmetric with respect to the sign of selection coefficient. However, the symmetry is limited to the case of constant size population and no dominance. In a growing population the mean ages of deleterious and beneficial alleles of the same frequency differ (see Methods). The assumption of constant population size greatly simplifies the analysis of allelic ages under a standard diffusion approximation. However, the assumption of constant population size is clearly violated for the human population. To investigate the case of a growing population, we resorted to forward computer simulations (see Methods for exact details of demographic history). Computer simulations indicated that the difference between mean ages of deleterious and neutral alleles of the same frequency is present in a recently rapidly expanding population, though it is smaller than in the case of a constant-size population (Figure 2A, 2B). The difference in ages was present also in a demographic scenario that included a bottleneck followed by a rapid recent population expansion (Figure 2C).

**Figure 3:**
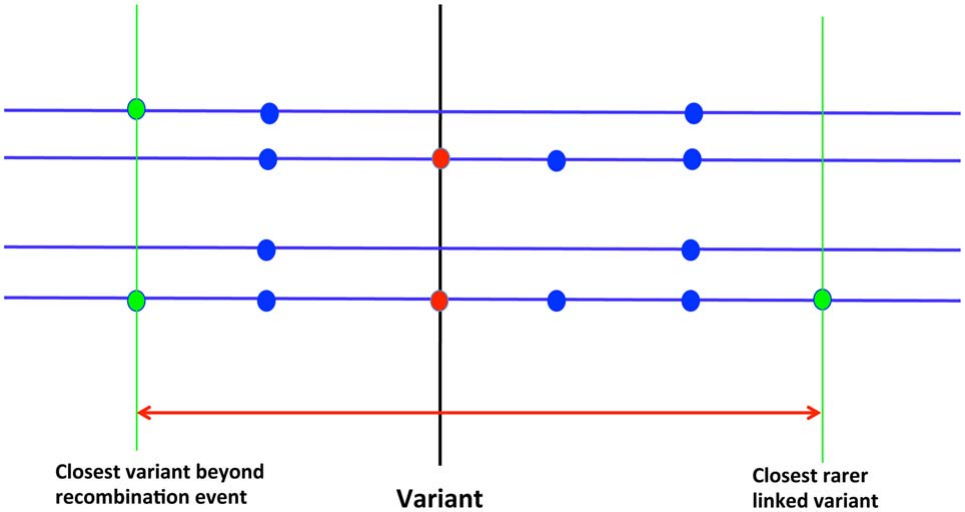
Cartoon presentation of the NC statistic. The NC statistic aims to capture the length of the haplotype carrying a variant. For a given variant (called the index variant, shown in the middle of the figure), the value of the NC statistic is the base-10 logarithm of the sum of physical distances measured up-stream (5′ direction) and down-stream (3′ direction) from the index variant to the closest variant that is either beyond a recombination spot (example shown on the left) or is linked to the index variant but is rarer than the index variant (example shown on the right). The red arrow in the figure illustrates that sum of the two distances.

We have developed a statistical approach to discriminate between classes of neutral and deleterious alleles at the same frequency. The test statistic, which we call the Neighborhood-based Clock (NC) is defined as the logarithm of the minimal physical distance to the nearest completely linked allelic variant at a lower frequency or to the nearest detectable recombination event (Figure 3). Therefore, younger alleles should correspond to larger values of the NC statistic. The intuition behind this statistic is that lower frequency allelic variants linked to the tested variant likely arose by mutation after the tested variant. Similarly, recombination events are expected to happen after introduction of the tested variant by mutation. The NC statistic captures information about the age of the alleles and especially about the time spent in the past at appreciable population frequencies.

To assess whether the NC statistic can indeed discriminate between functional classes of human allelic variants, we analyzed coding variants discovered in the pilot data from the Genome of Netherlands project (GoNL). The pilot GoNL dataset (see Methods) consists of complete genomes of 47 parent-child trios, which enables accurate variant calling and haplotype phasing. Thus, the unique trio-based design of the GoNL dataset allowed us to compute NC statistics informed by family-based rather than population-based phasing, an especially important advantage for rare and low frequency alleles.

**Figure 4:**
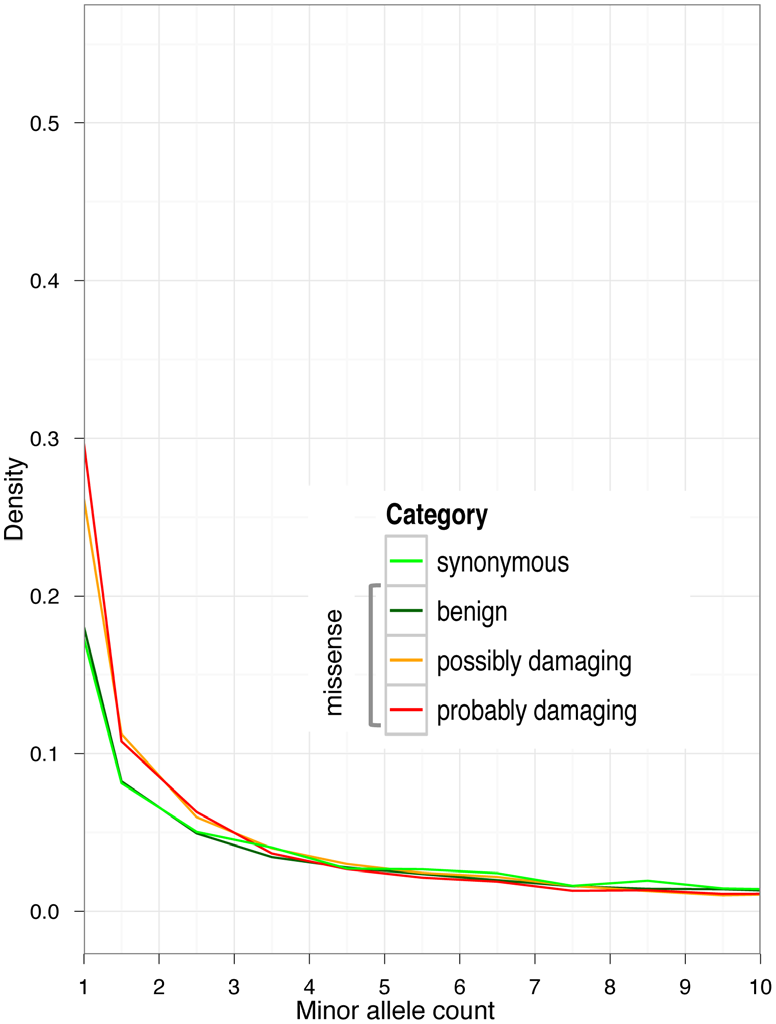
Allele frequency spectra in GoNL data, for synonymous alleles and non-synonymous alleles stratified by PolyPhen-2 functional predictions. For better presentation, the graphs have been cropped at minor allele count 10.

We subdivided all coding variants into synonymous and non-synonymous (missense and nonsense). We further annotated the missense variants using PolyPhen-2 predictions as benign, possibly damaging, and probably damaging [15]. In the GoNL dataset, consisting of 94 unrelated parents, there are 25997 common coding SNPs with a minor allele count 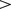
20. Of those common SNPs, 13956 (53.7%) are synonymous and 12041 (46.3%) are non-synonymous (including 1466, or 5.6%, of probably damaging missense SNPs). The fraction of non-synonymous and, especially probably damaging SNPs, increases for SNPs with low frequencies (Figure 4). At minor allele count 2 there are 7437 coding SNPs, of which 3102 (41.7%) are synonymous, and 4335 (58.2%) are non-synonymous (including 1176, or 15.7%, of probably damaging missense SNPs).

We estimate that 14.8% of non-synonymous alleles at minor allele count 2 are deleterious. At minor allele count 2 there are 3102 synonymous SNPs (which constitute 7.9% of all 39454 synonymous SNPs). In contrast, at minor allele count 2 there are 4335 non-synonymous alleles (which constitute 9.2% of all 46946 non-synonymous SNPs). Therefore, there is an enrichment of rare non-synonymous alleles compared to synonymous alleles. If we assume that all synonymous SNPs are selectively neutral, then we can treat their distribution as the neutral expectation. Therefore, there are 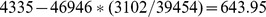 more non-synonymous alleles at minor allele count 2 than expected if all non-synonymous variants were neutral. Those 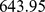 alleles constitute 14.8% of all 4335 non-synonymous alleles at minor allele count 2. By the same logic, we estimate that 27% of probably damaging missense variants at minor allele count 2 are deleterious.

Below, we focus primarily on low-frequency derived alleles (i.e., alleles that differ from the ancestral state). We note that, even though the theoretically predicted difference in age is greater for high-frequency deleterious variants (Figure 1b), we expect that the difference between functional categories of coding variants can be detected only for variants with derived allele frequency up to 10%, because deleterious variants rarely ever reach higher frequency.

**Figure 5:**
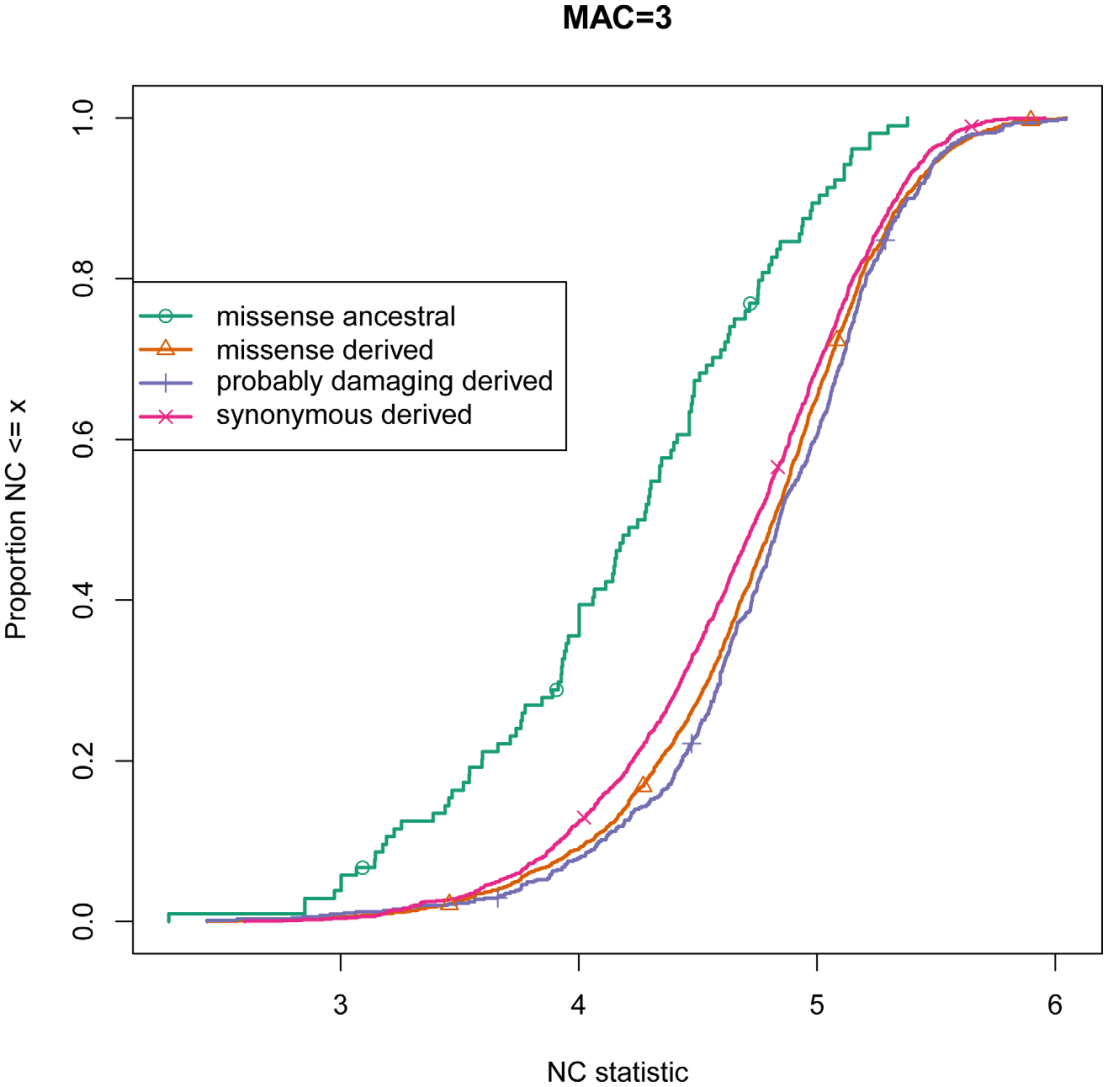
Empirical Cumulative Distribution Function of the NC statistic for alleles at minor allele count 3 in GoNL data. Synonymous derived variants serve as the baseline distribution. The distribution of NC for probably damaging derived missense variants is notably shifted towards higher values, consistent with their younger age. The NC-statistic distribution for ancestral alleles are at minor allele count 3 is strongly shifted towards lower values, consistent with much older age of those alleles.

**Figure 6:**
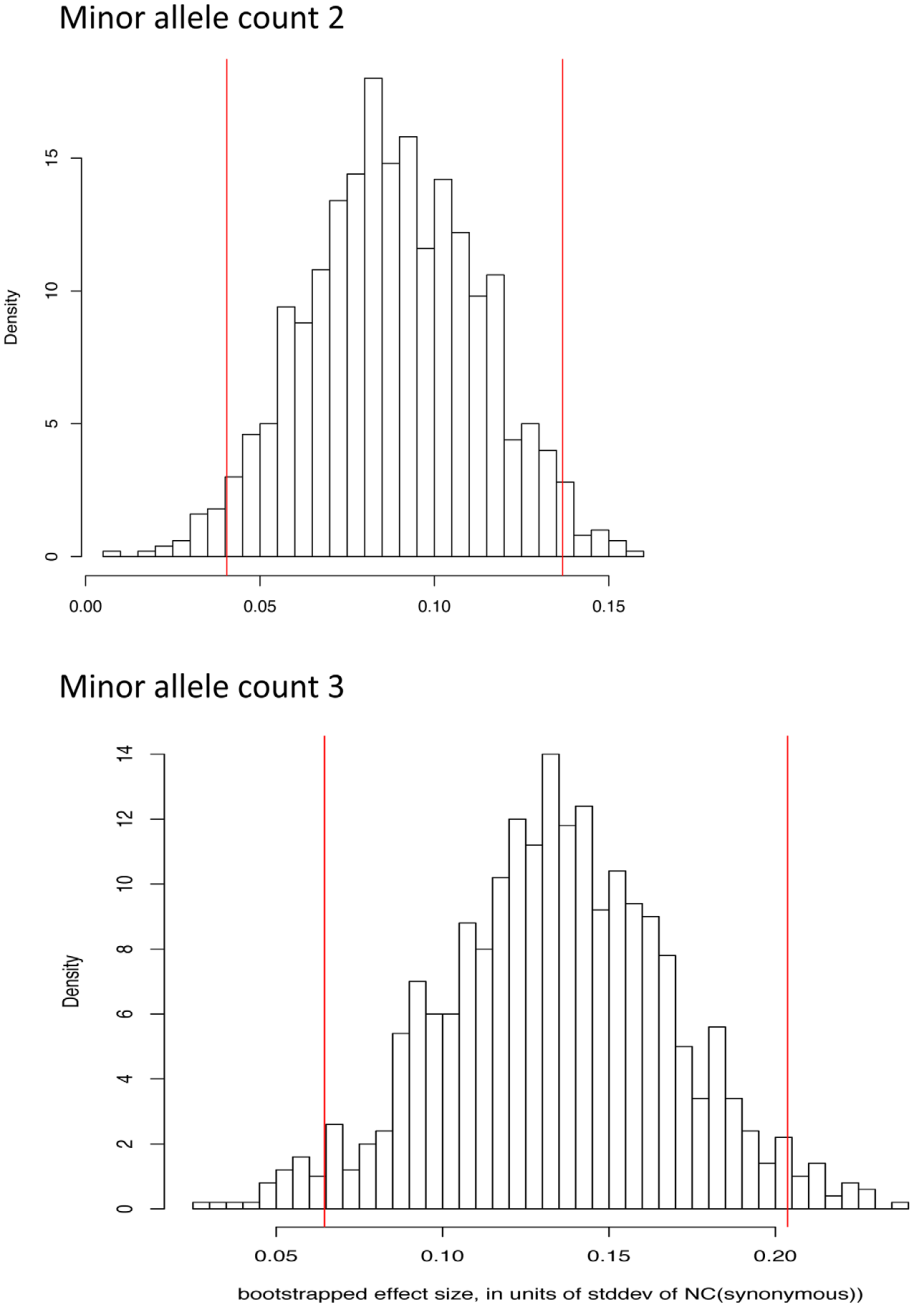
Bootstrap distribution of normalized difference between NC statistic on missense and synonymous variants for derived allele count 2 and 3. Vertical red bars indicate 95% confidence intervals. For presentation, panels have been aligned along the X axis.

The NC statistic can discriminate between non-synonymous and synonymous SNPs at the same derived allele frequency (Figure 5 and Table 1) and bootstrap analysis shows that the effect is not explained by a small number of variants (Figure 6). This is consistent with the abundance of low frequency deleterious non-synonymous alleles in humans. Variants predicted to be probably damaging by PolyPhen-2 have higher values of NC statistics. Overall, we observe a positive correlation between PolyPhen-2 predictions of damaging effects of derived missense variants and the NC test statistic (Table 2). This result indicates that the NC statistic independently captures some of the same selective characteristics of variants as PolyPhen-2, and it may contain additional signal not present in the conservation or structural properties which PolyPhen-2 is based on.

**Table 1:** Discrimination of derived missense alleles by the NC statistic.

MAC	Variants	N	meanNC	Effect size	95% CI	P
2	coding-synon	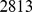	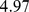	baseline		
2	missense	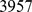	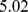	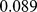	( 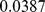 , 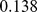 )	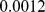
2	benign	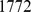	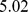	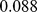	( 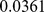 , 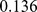 )	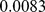
2	possibly damaging	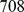	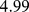	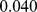	( 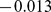 , 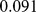 )	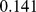
2	probably damaging	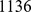	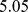	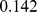	( 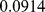 , 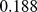 )	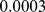
3	coding-synon	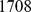	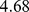	baseline		
3	missense	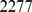	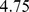	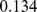	( 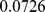 , 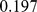 )	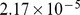
3	benign	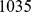	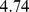	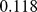	( 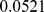 , 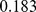 )	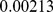
3	possibly damaging	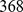	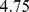	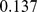	( 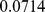 , 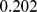 )	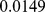
3	probably damaging	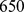	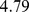	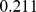	( 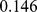 , 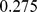 )	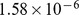
4	coding-synon	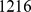	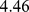	baseline		
4	missense	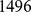	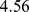	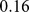	( 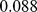 , 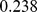 )	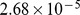
4	benign	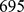	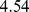	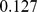	( 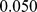 , 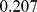 )	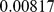
4	possibly damaging	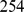	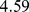	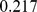	( 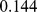 , 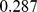 )	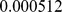
4	probably damaging	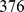	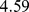	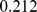	( 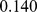 , 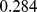 )	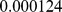
5	coding-synon	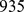	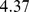	baseline		
5	missense	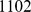	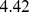	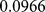	( 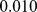 , 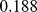 )	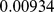
5	benign	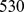	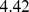	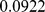	( 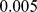 , 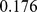 )	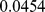
5	possibly damaging	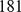	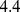	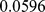	( 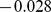 , 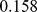 )	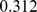
5	probably damaging	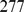	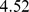	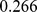	( 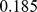 , 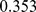 )	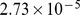
6	coding-synon	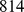	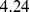	baseline		
6	missense	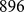	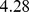	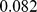	( 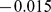 , 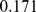 )	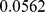
6	benign	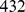	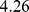	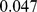	( 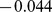 , 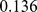 )	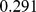
6	possibly damaging	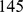	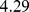	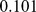	( 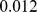 , 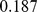 )	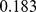
6	probably damaging	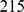	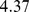	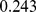	( 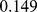 , 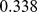 )	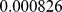
2–6	coding-synon	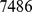		baseline		
2–6	missense	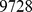				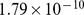
2–6	benign	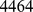				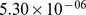
2–6	possibly damaging	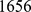				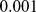
2–6	probably damaging	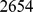				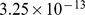

Missense alleles are sub-classified info categories based on *PolyPhen-2* predictions. Effect sizes were calculated as standard deviations from the mean of the NC statistic for synonymous variants at the same minor allele count (MAC). Within each MAC class, P-values were calculated by 1-sided Mann-Whitney test. Combined P-values for MAC 2–6 were computed by meta-analysis (Methods).

**Table 2:** Correlation between the NC statistic and PolyPhen2 predictions.

	derived			ancestral		
MAC	N	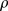	P	N	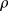	P
2	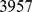	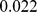	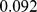	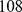	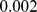	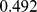
3	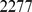	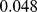	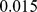	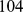	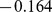	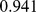
4	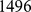	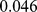	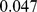	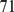	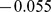	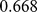
5	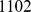	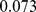	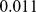	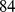	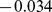	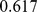
6	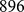	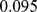	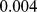	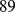	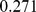	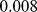

Within each minor allele count, derived missense alleles are positively correlated (Spearman's 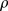) with PolyPhen2 predictions (pph2_prob), while no such correlation exists for ancestral missense alleles. P-values are 1-sided (alternative hypothesis 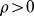).

Low-frequency ancestral alleles are expected to be much older than derived alleles at the same minor allele frequency. Those ancestral alleles date from before the human-chimpanzee divergence and each low-frequency ancestral allele corresponds to a high-frequency (i.e., almost fixed) derived allele. For example, an ancestral allele at minor allele frequency of 1% corresponds to a derived allele at population frequency of 99%. In agreement with this expectation, the NC statistic is, on average, much lower for ancestral variants than for derived variants (Figure 5).

**Figure 7:**
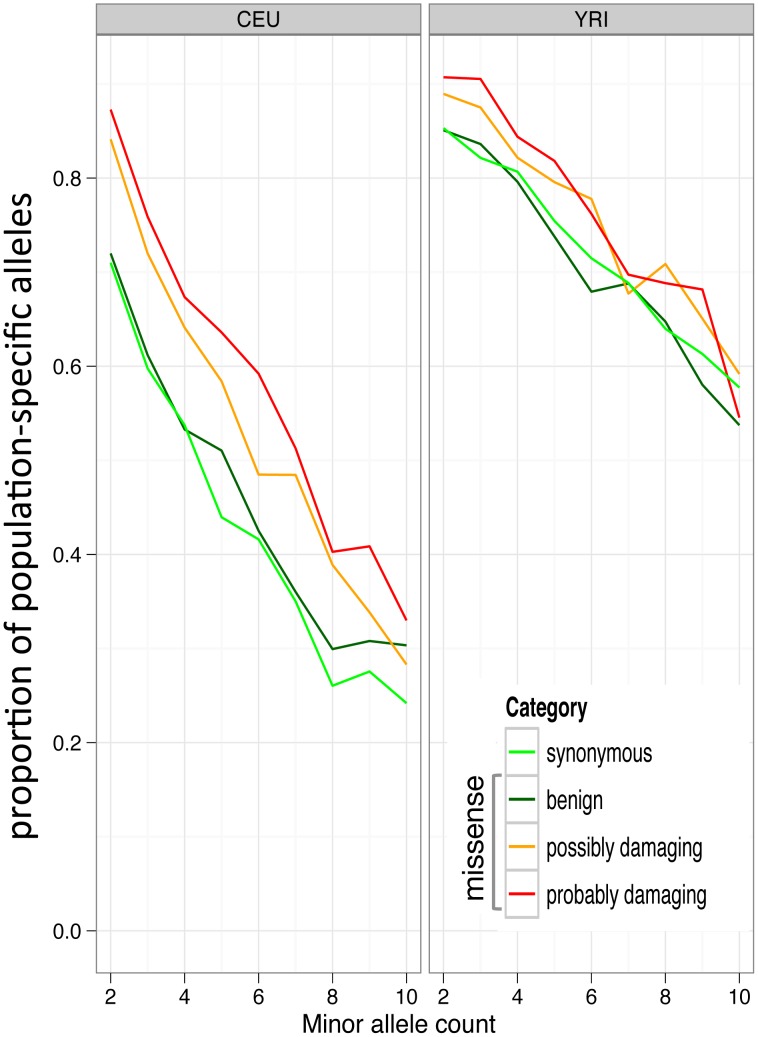
Allele frequency spectra and population-private coding alleles. The graphs show the proportion of population-private synonymous alleles and non-synonymous alleles stratified by PolyPhen-2 functional predictions.

As another independent test whether deleterious variants are on average younger than neutral alleles of the same frequency, we analyzed the fraction of population-specific SNPs. Because this analysis required data from multiple human populations, we used an entirely different data set, pilot data from the 1000 Genomes project (see Methods). We observed that non-synonymous SNPs, especially those predicted to be damaging, are more often population-specific (Figure 7) than synonymous SNPs of the same frequency. This is consistent with non-synonymous SNPs being on average younger. As expected, the difference disappears at population frequencies greater than 10%. Previously, also using 1000 Genomes data, Marth *et al.* [16] showed an increase in population specificity of variants in coding regions compared to intergenic regions. Importantly, this analysis is independent of the NC statistic and of the GoNL data, and thus provides additional evidence of the younger age of deleterious alleles.

Finally, we examined examples of published low frequency variants shown to be significantly associated with human complex traits. Variants R46L of *PCSK9* associated with reduction of LDL-cholesterol [17] and two variants in *IFIH1* (I923V and H460R) associated with Type-I diabetes [18] have been observed in the GoNL dataset. The *PCSK9* R46L variant and *IFIH1* I923V variant are both younger than average according to the NC statistic (33rd and 9th percentile, respectively). The *IFIH1* H460R variant is a low-frequency ancestral allele and, accordingly, has low NC statistic (indicating old age), at 2.4 standard deviations lower than average for synonymous variants at the same minor allele count (lower than 99.2% of synonymous variants at allele count 4). These results suggest that although the NC statistic cannot be applied to pinpoint individual functional variants (at least in relatively small sequencing datasets available at present), it may have potential to enrich for groups of functional variants in burden association tests (reviewed in [19]). This must be investigated in the future on much larger datasets.

Our approach does not distinguish effects of positive and negative selection. As noted above, the theory predicts that the effect of selection on age and on time spent at each frequency in the past is symmetric with respect to selection coefficient, assuming no population growth and no dominance effects (in a quickly growing population strong positive selection produces younger alleles than negative selection [20]). We focused on negative selection in this study because at low derived population frequencies many missense variants are deleterious [5] and very few are advantageous. Nonetheless, our approach may be applicable to positive selection too.

Our analysis benefitted from whole-genome sequencing data allowing low-frequency alleles far away from the coding regions (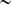
100 kb) to be identified. Additionally, the accurate haplotype phasing available in the trio-based sequencing data from GoNL was indispensable for our analysis, which required accurate identification of linked variants and recombination events.

To our knowledge, ours is the first large-scale real-data analysis of this effect theoretically predicted by Maruyama in 1974. Our analysis provides additional evidence, completely independent of allele-frequency distribution, for the abundance of deleterious alleles in coding regions in the human population.

## Methods

Theoretical mean ages and sojourn times were computed for constant-size populations, using diffusion approximation of the stochastic process. Let 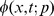 be the probability density that the allele frequency in the 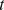 th generation is between 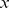 and 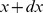, 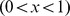 given its starting frequency 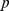. Then, 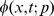 satisfies the backward Kolmogorov equation
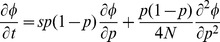
Following Maruyama and Kimura [21], we denote by
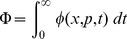
the density of mean sojourn time at frequency 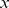 starting at frequency 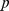 before fixation or loss. Then, 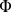 satisfies the equation
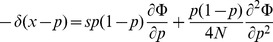
where 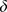 denotes Dirac's delta function.

Now, given the current frequency 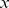 and initial frequency 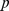, the density of mean sojourn time at frequency 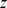 is
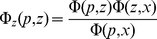
For the boundary conditions, for all 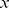, 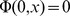 and 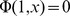, the density for frequency 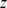 below 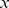 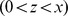 is
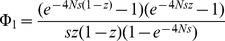
while the density at frequency 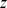 above 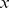 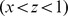 is
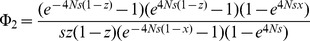
It then follows that mean age of a variant at current frequency 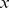 is the sum of sojourn times at all frequencies
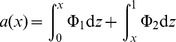
Both the sojourn times and age are symmetric functions of the selection coefficient 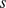. In other words, deleterious and advantageous alleles at a given frequency are expected to be younger than neutral alleles, and selected alleles are expected to spend progressively less time at higher frequencies leading to the current population frequency.

Forward-in-time, individual-based computer simulations were performed in SFS_code [22]. The parameters were selected to examine the behavior of the age of selected alleles and not to emulate realistic demographic scenarios. Coding region of 100 kb or 200 kb was simulated for 2.05 N generation after the initial burn-in of 10 N generations (N = 5000 or 10000). 70% of simulated variants were under selection, the remainder were neutral. Expansion phase started at time 2 N generations after burn-in, with expansion rate of 156.48. The scaled mutation rate per site was 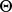 = 4N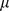 = 0.0001, and scaled recombination rate per site 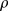 = 4Nr = 0.0001. Additionally, a scenario that included a bottleneck was simulated. The bottleneck was an instantaneous population reduction of 50% at time 2N, followed by rapid population expansion as in other simulation scenarios.

The data presented here include SNP genotypes in a pilot subset of 47 trios collected by the Genome of the Netherlands (GoNL) Project (http://www.nlgenome.nl), using whole-genome sequencing at 12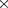 coverage with Illumina HiSeq technology performed at Beijing Genome Institute (BGI). The sequence data were aligned to the human reference genome build hg19 using BWA [23], duplicate reads removed, re-alignment performed around insertions/deletions from the pilot of the 1000 Genomes Project [24], and base quality scores recalibrated. Variant discovery and genotyping was done using the Unified Genotyper in the Genome Analysis Toolkit (GATK) [25] across all individuals simultaneously. The initial calls were filtered using Variant Quality Score Recalibration (VQSR) [26], resulting in 11,521,751 biallelic SNPs identified with a corresponding Ti/Tv ratio of 2.21. We used Phase By Transmission in the GATK to calculate the posterior probability for all possible genotypes in each trio from the raw genotype likelihoods and expected modes of transmission, and identified the best-guess genotype in the trios. We phased these best-guess SNP genotypes for all trios using Beagle v3.3 [27].

Data from July 2010 release of the 1000 Genomes low-pass pilot data was used. Variant annotations and functional predictions were computed using PolyPhen-2. In all analyses, only non-singleton variants (i.e., with minor allele counts at least 2) were used and only those that had annotated phased genotypes.

The NC test statistic was computed for variants at minor allele count of 2–6 separately. The statistic, for each coding variant, was computed as base-10 logarithm of the sum of the up- and down-stream physical distances to the closest recombination event (computed using the 4-gamete test [28]) or a fully linked rarer variant, i.e., variant present on a strict subset of the haplotypes.

The ancestral/derived states of variants were calculated using the ancestral reference human_ancestor_GRCh37_e59 provided with the 1000 Genomes project.

P-values were computed using Mann-Whitney rank-sum test. P-values were 1-sided, with alternative hypotheses following younger age for non-synonymous variants. Effect sizes were calculated as standard deviations from the mean of the NC statistic for derived synonymous variants at a given minor allele count. Confidence intervals were computed using the percentile bootstrap method on 1000 bootstrap permutations of variant labels. Combined p-values were computed by meta-analysis using the Z-score method, weighted by sample size.
